# Protective Factors Enhancing Prosocial Behavior and Preventing Internalizing and Externalizing Symptoms among Adolescents Living in Forster Care Homes

**Published:** 2014

**Authors:** Maria E. Aguilar-Vafaie, Mehrnoosh Roshani, Hamidreza Hassanabadi

**Affiliations:** 1 Professor, Department of Psychology, Tarbiat Modares University, Tehran, Iran.; 2 Department of Psychology, Tarbiat Modares University, Tehran, Iran.; 3 Department of Educational Research, Tehran University, Tehran, Iran.

**Keywords:** Orphan, Prosocial Behavior, Protective Factors, Residential Foster Care, Resilience

## Abstract

**Objective**: Based on Problem Based Theory, this study investigated a broad array of putative protective factors associated with psychopathological symptoms and prosodical behaviour.

**Methods:** Participants were 140 orphan adolescent girls and boys living in foster care homes in Tehran, chosen with convenience sampling procedures. Using a cross-sectional design this study examined the individual and interactive properties of protective factors in this high-risk population.

**Results:** Findings with theoretically derived multi-item subscales indicated a high degree of association specificity based on type of psychopathology and depending on gender. Results with the whole sample indicated that theoretically derived individual protective factor scales associations were obtained mainly for conduct problems and emotional symptoms, and with girls only.

**Conclusion:** The present study provides introductory information on the identification of protective factors that can be utilized in educational, interventional and preventive public health programs for this high-risk population. One innovative contribution of the present research is to provide an introductory validation of a theory-based model of adolescent protection and resilience, for which there is ample empirical support, in a high-risk population of Iranian adolescents living in foster homes centers in a metropolitan urban setting.

## Introduction

Published statistics about children in out-of-home care in the Islamic Republic of Iran (IR Iran) indicate that for the year 2010 about 1,150,000 are expected to enter the governmental child welfare centers throughout the country (1). In addition, the percentage of orphans due to HIV infection as percentage of the total orphan population has had an increasing profile from 0.0 in 1990 to 0.5 in 2005 and a projected 2.9 in the year 2010 (1).

Furthermore, the situation of orphans in the IR Iran is such that due to cultural barriers against adoption, these children do not achieve stability (or permanence) as a consequence of not becoming adopted in a relatively reasonable short period of time after entering the welfare care system; rather, these children may stay in these centers for long periods of time of more than 20 years. Statistics on these facts are difficult to obtain as they are not usually made public.

Despite these statistics, systematic research, especially longitudinal studies on foster care populations, is lacking (2) and what seems most distressing is that there seems to be no awareness at higher levels of governmental administration of the necessity of longitudinal research in this regard (3). What is known about children entering foster care is that they have had histories of maltreatment and neglect due to addiction or mental illness of one or both of their parents (3). In addition to suffering the consequences of child maltreatment and neglect, children placed in foster care centers are likely to experience additional trauma by being removed and often isolated from their homes, schools, friends and family. Furthermore, these stressors may be exacerbated by frequent placement changes, not uncommon for older foster children (2). Given their already increased risk of emotional and behavioral problems, youth in foster care may lack appropriate coping resources to handle the multiplicity of stressors associated with multiple life transitions. Not surprisingly, maltreated and neglected children in foster care are overrepresented among multiple service systems including juvenile justice and mental health. Evidence is also beginning to consolidate regarding the specificity of girls’ psychopathology in terms of etiology, risk and protective factors (4), such that factors associated with resilience may not be the same for both genders.

The operationalization of protective factors has been a source of controversy. In recent years, this term has been used to refer to all factors associated with positive outcomes, regardless of whether relationships are stronger for children living in high-risk contexts (5). Luthar and colleagues (6) argue that while interaction effects provide useful knowledge on the processes that function specifically under conditions of risk, main effects can also be informative. For example, in designing interventions for at-risk children, addressing any and all factors that attenuate the effects of risk are likely to be beneficial.

Implicit within this controversy is the issue of what type of sample is optimal for studying resilience. If the goal is to simply identify protective factors that help children at high levels of risk, regardless of their impact at other levels of risk, a low risk subgroup is unnecessary. 

Beyond these basic problems of definition, at the present, there appears to be an unanimous consensus among researchers regarding the usefulness of the identification and promotion of resilience and health through awareness and education about factors that promote positive development rather than focusing on risk factors (7). Moreover, Hawkins and colleagues (4), who are experts in the field of positive youth development, indicate that in view of the limitations of risk focused intervention strategies, research on resilience (8, 9) turned toward protective factors-aspects of individuals and their environments that buffer or moderate the effect of risk are more advantageous. 

Protective factors have been identified in three main areas: within the child, within the family, and within the community. A full discussion on widely researched protective factors is beyond the scope of this article, however in order to become familiarized with the area; the reader is advised to consult more comprehensive reviews (10-12).

Problem Behavior Theory (PBT) (13, 14) proposes three composite measures of protection—models, controls, and support—at the individual level and at levels of the adolescents’ foster home, peer group, school and neighborhood and hypothesize that measures of protection will be negative predictors of internalizing and externalizing problems. Conversely, these protective measures are expected to be positive predictors of prosocial behavior. Therefore, one innovative contribution of the present research is to provide a theory-based model of adolescent protection and resilience within the framework of positive youth development for a high-risk population of Iranian adolescents living in foster homes centers in a metropolitan urban setting.

## Materials and Methods

***Participants***

Participants were 140 orphan adolescent girls (n = 69) and boys (n = 71) with the mean age of 15.4 ± 1.54 years (range 11–18) from governmental foster home centers in Tehran, mainly representing eastern and southern city district areas. Through convenience sampling method, three groups were represented: early adolescence (between 11 and 13 years old; 21 females and 17 males), middle adolescence (between 14 and 15 years old; 32 females and 33 males) and late adolescence (between 16 and 18 years old; 16 females and 21 males).

***Procedures***

The study was described as an investigation of adolescent mental health. Foster home consent was obtained, wherein an informed consent letter was sent to each foster home center after obtaining permission from the Ministry of Health. Participants completed a risk factor survey in their own classrooms and foster-care caregivers filled out questionnaires for each adolescent regarding their mental health. The presence of multi-caregivers—the person who attends the adolescent at the foster home—is the norm in residential foster care. Strengths and Difficulties Questionnaire (SDQ) ratings were provided by the adolescents’ caregivers at the foster homes. 

***Measures***

*Protective Factors*

Five multi-item subscales conformed the models protection composite (23) assessing caregivers’ expectations for the adolescents’ academic achievement or MP-H (e.g., “Is it important for your teachers that you do well at school, obtain a diploma and be able to advance into college?”; α = 0.80; peer disapproval of deviant behavior or MP-P (e.g., “What is the opinion of most of the students in your school about cheating in exams?”; α = 0.86); classmates’ disapproval of use of drugs at school or MP-S (e.g., “What is the opinion of most of the students in your school about smoking?”; α = 0.79); adults disapproval of deviant behavior and use of drugs/alcohol or MP-NO (e.g., “What do you think is the opinion of adults in your neighborhood about stealing the property of others?”; α = 0.81); and, adults in the neighborhood reactions to social deviance or MP-NA (e.g., “If adults in your neighborhood see your friends damaging the property of others or using marijuana/hashish/opium do they try to stop them?”; α = 0.82).

Five multiple item subscales conformed the controls protection composite and assessed control based protection at the individual, home, peer levels, as follows: the adolescents’ judgment on the detrimental effects of drugs on health or CP-D (e.g., “Does smoking can have an effect on your health?”; α = 0.86); the adolescent’s beliefs about health behaviors or CP-B (e.g., “To what extent do you think it is correct to continuously exercise or have a systematic schedule for doing exercise?”; α = 0.64); caregivers monitoring at the foster home center or P-H (e.g., “In the place where you live how strictly are rules observed?”; α = 0.66); peers’ sanctions for deviant behavior or CP-P (e.g., “What is your friends opinion about you bullying your friends?”;α = 0.74); and, teachers’ sanctions for deviant behavior or CP-T (e.g., “Do you get in trouble if your teachers see you stealing from a store, even if it is a minor thing?”; α = 0.73(.

The support protection composite index was composed of nine multi-item scales at the individual, home, peers, school and community levels, as follows: adolescents’ beliefs about antisocial behavior, smoking and use of illicit drugs or SP-AD (e.g., “To what extent do you think is wrong to tell lies to your caregiver about something you did?”; α = 0.81); adolescents’ positive attitude toward school or SP-AS (e.g., “How much do you agree with this statement: I like to go to school”; α = 0.70); caregivers support or SP-HS (e.g., “Do your caregivers’ show interests in enjoyable after-school activities, or activities during holydays and summer vacation?”; α = 0.74); caregivers’ monitoring or SP-HM (e.g., “How accurately and strictly is sleep time kept in your foster home?; α = 0.54”); caregivers’ encouragement for prosocial behavior or SP-PS (e.g., “In the place where you live, do your caregivers encourage you to play sports and to study?”; α = 0.86); peer support or SP-P (e.g., “Do your friends pay attention to your opinions and interests?”; α = 0.53); caring and nurturing social climate at school or SP-S (e.g., “Do your teachers at school behave in such a way that you feel respected?”; α = 0.72); adolescents’ feeling of intimacy and connectedness with teachers/adults or SP-I (e.g., How much do you agree with this statement: It is enjoyable for me when I do things with my teachers”; α = 0.86); and, adolescents’ perceived neighborhood efficacy or SP-NE (e.g., “Do people in your neighborhood help and take care of each other?”; α = 0.82).

*Adolescent Religiosity*

An 11-item, 3-factor scale was designed for this study to measure self-reported degree of spirituality and religiosity of adolescents. A 4-point Likert scale was used with categories of 1: almost never, 2: sometimes, 3: often, and 4: almost always. The first factor [reliability coefficient of internal consistency (α) = 0.82, mean = 13.84 ± 2.58, range: 4-16] included 4 items denoting the importance of religion and a positive personal relationship of the adolescent with God (e.g., “I feel the presence of God in my life”); Factor 2 (α = 0.70, mean = 10.05 ± 2.86, range: 4-16) consisted of four items, and indicated importance of praying for the adolescent; and Factor 3 (α = 0.73, mean = 5.16 ± 1.72, range: 2-8) consisted of two items (e.g., “In my opinion appropriate dressing should be made necessary for both sexes”), denoting the importance of religious-based social values and conventions.

*Strengths and Difficulties Questionnaire (SDQ)*

The standard SDQ (16) proposes a five-factor structure for the assessment of adolescents’ behavioral and emotional problems as well as behavioral strengths, such that it includes four problem subscales (emotional symptoms, conduct problems, hyperactivity/inattention, and peer problems) and one prosocial behavior scale. Each item is rated on a 3-point Likert scale ranging from 0 (not true), 1 (somewhat true), or 2 (certainly true) and each of the SDQ subscales consists of five items, thus yielding scores between 0 and 10. Alpha coefficients of internal consistency were computed for all standard problem behavior subscales (Conduct problems, α = 0.60 (mean = 2.96 ± 2.17); Attention deficient and hyperactivity disorder (ADHD), α = .64 (mean = 4.20 ± 2.28); Emotional symptoms, α = 0.61 (mean = 3.20 ± 2.10); and peer problems, α = 0.44 (mean = 3.06 ± 1.95), and prosocial behavior, α = 0.72 (mean = 6.64 ± 2.50).

***Statistical analysis***

Data handling and all statistical analyses were carried out using SPSS software (Statistical Package for the Social Sciences; release 17.0). The present study utilized hierarchical regression analysis as this approach permits each protective factor subscale to be optimally weighted in the regression equation to maximize the criterion variance accounted for, in contrast to the equal weighting that each risk factor item has in its composite measure. 

## Results

Pearson product correlations coefficients, means, and standard deviations for measured variables at the composite, theoretically derived indices, and subscale levels are presented in table 1.

Findings regarding the theoretically predicted relationship between prosocial behavior and protective factors are depicted in table 2.

Table 3 depicts results on the prediction of separate SDQ difficulties subscale scores by protection subscales scores.

Analyses of each gender separately regarding the prediction of separate SDQ difficulties subscale scores by support subscales scores are depicted in table 4.

## Discussion

To begin with, assessment of descriptive statistics on major variables of the research indicate that SDQ total difficulties scores of adolescents in the present sample felt within the borderline classification based on normative data of Iranian children (17), and about 25% of adolescents in the sample felt within the clinical cut-off, supporting the ‘at risk’ status of the present sample. Similar ‘at risk’ diagnosis can be made in relation to prosocial behavior, with about 30% of the adolescents in the sample falling within the clinical cut-off (17).

When the SDQ criterion is decomposed into specific disorders, and composite protection indices are decomposed into protective factor subscales, significant predictors were obtained for conduct problems and emotional symptoms in the whole sample, indicating a selective pattern of protective factors association with psychopathological symptoms and with gender. Such that, in case of the whole sample, for conduct problems only one main effect was obtained, adherence to religious motivated social norms, along with three gender interactions: adolescents’ positive attitude toward school; adolescents’ feelings of intimacy and connectedness with teachers; and peer disapproval of deviant behavior. For emotional symptoms, mainly gender interaction effects were significant. The gender interactions indicate that some effects are stronger for one gender than for the other and that Gender x Protection interactions are relatively strong, accounting for a significant increment of amount of variance of 18% and 19%, respectively. The present findings, involving the association between religious motivated adherence to social norms and conduct problems is relevant to the role of religion in adolescents’ development. Some literature points to the limiting protective effect of religion suggesting that religion only protects against minor offense (18). However, despite the lack of consensus in the field regarding the impact or religiosity on different types of delinquent behaviors, research has established that religion does, in fact, have some influence on some delinquent behaviors (19, 20).

**Table 1:** Means, standard deviations and correlations between covariates, protective factors and outcomes (n = 140)

**Variables**	**1**	**2**	**3**	**4**	**5**	**6**	**7**	**8**	**9**	**10**	**11**	**12**	**13**	**14**	**15**	**16**	**17**	**18**	**19**	**20**	**21**	**22**
**1. MP-C**	-																					
**2. MP-H**	0.473**	-																				
**3. MP-P**	0.717**	0.121	-																			
**4. MP-S**	0.676**	0.092	0.384**	-																		
**5. MP-NO**	0.714**	0.174*	0.364**	0.214**	-																	
**6. MP-NA**	0.305**	0.224	0.143	0.221**	0.208**	-																
**7. CP-C**	0.037	-0.033	0.031	-0.025	0.105	0.028	-															
**8. CP-D**	-0.102	-0.227**	-0.063	-.071	0.050	0.017	0.691**	-														
**9. CP-B**	-0.017	-0.072	-0.022	0.063	-0.029	0.222**	0.425**	0.208**	-													
**10. CP-H**	-0.036	-0.037	-0.028	-0.057	-0.030	-0.025	0.619**	0.234**	0.094	-												
**11. CP-P**	0.278**	0.131	0.196*	0.125	0.262**	0.292**	0.511*	0.154	0.050	0.179*	-											
**12. CP-T**	0.045	0.097	0.046	-0.068	0.061	0.024**	0.614**	0.172*	0.146	0.248**	0.193*	-										
**13. SP-C**	0.121	0.252**	-0.014	0.161	-0.045	0.223**	0.190*	-0.013	0.074	0.054	0.442**	0.078	-									
**14. SP-AD**	0.011	0.165	-0.034	0.034	-0.097	-0.010	0.010	-0.055	0.100	0.122	0.183*	-0.002	0.564**	-								
**15. SP-AS**	0.195*	0.254**	0.134	0.110	0.047	0.152	0.317**	-0.025	0.167*	0.268**	0.417**	0.138	0.624**	0.187*	-							
**16. SP-HS**	0.172*	0.309**	-0.004	0.157	0.025	0.214*	0.077	-0.061	-0.012	0.014	0.213*	0.109	0.656**	0.216*	0.200*	-						
**17. SP-HM**	0.063*	0.059	0.000	0.067	0.037	0.224**	0.112	0.019	-0.100	0.030	0.259**	0.108	0.462**	0.036	0.097	0.534**	-					
**18. SP-PS**	0.167*	0.060	0.011	0.030	0.210*	0.040	0.106	-0.010	0.028	0.062	0.295**	0.010	0.609**	0.236**	0.313**	0.423**	0.400**	-				
**19. SP-P**	0.130	0.158	0.062	0.302**	-0.071	0.191*	0.110	0.040	-0.100	0.030	0.210*	0.130	0.353**	-0.070	0.303**	0.178*	0.093	0.010	-			
**20. SP-S**	0.070	0.054	-0.044	0.124	0.040	0.189*	0.116	-0.024	-0.061	0.119	0.308**	0.021	0.614**	-0.008	0.488**	0.274**	0.278**	0.343**	0.490**	-		
**21. SP-I**	-0.033	0.113	-0.107	0.041	-0.107	0.203*	0.232**	0.116	0.120	0.136	0.278**	0.059	0.678**	0.123	0.484**	0.446**	0.291**	0.433**	0.210*	0.383**	-	
**22. SP-NE**	-0.011	0.049	-0.021	0.080	-0.120	0.196*	0.069	-0.010	0.076	-0.060	0.299**	0.040	0.657**	0.164**	0.367**	0.306**	0.200*	0.373**	0.350**	0.513**	0.492**	-
**23. R-1**	0.003	0.157	0.034	-0.047	-0.088	0.110	0.077	-0.114	-0.059	0.150	0.108	0.162	0.429**	0.202*	0.396**	0.288*	0.143	0.158	0.150	0.251**	0.311**	0.334**
**24. R-2**	-0.109	0.087	-0.092	-0.128	-0.113	-0.020	0.096	0.027	-0.119	0.173*	0.019	0.127	0.139	-0.036	0.312**	-0.014	0.027	-0.050	0.190*	0.259**	0.019	0.179*
**25. R-3**	0.073	0.062	0.094	-0.036	0.078	0.119*	0.084	0.027	-0.086	0.037	0.211*	0.044	0.201*	-0.071	0.264**	0.051	0.100	0.113	0.250**	0.310**	0.149	0.219**
**26. SDQ-TD**	-0.090	-0.210	-0.076	0.007	-0.037	-0.076	-0.039	0.042	0.000	-0.085	0.020	-0.098	0.051	-0.006	0.100	-0.088	0.014	0.039	0.000	0.155	0.077	0.032
**27. SDQ-CP**	-0.012	-0.100	0.017	0.039	0.019	-0.152	-0.131	-0.026	-0.033	-0.033	-0.125	-0.176*	-0.056	-0.191*	0.074	-0.105	0.010	0.071	0.091	0.166	-0.089	0.090
**28. SDQ-ADHD**	-0.072	-0.255**	-0.077	0.049	-0.020	-0.062	0.098	0.109	0.026	-0.013	0.143	0.013	0.105	0.020	0.168*	-0.046	-0.053	0.089	0.043	0.174*	0.178*	0.097
**29. SDQ-EP**	-0.192*	0.066	-0.165	-0.098	-0.162	-0.120	-0.093	0.022	0.019	-0.237**	-0.028	0.044	0.160	0.219**	0.038	0.010	0.098	0.109	-0.127	0.089	0.115	0.047
**30. SDQ-PP**	0.040	-0.033	0.026	0.027	0.068	0.006	0.016	0.001	-0.013	0.059	0.062	-0.056	-0.084	-0.019	0.031	-0.096	-0.021	-0.031	-0.123	-0.029	-0.009	-0.171*
**31. SDQ-PS**	0.044	0.200*	0.011	0.072	-0.121	0.178*	-0.064	-0.167*	0.048	-0.195*	0.113	0.097	0.299**	0.358**	0.041	0.248**	0.083	0.226**	-0.036	0.012	0.157	0.155
**Mean**	31.56	7.76	7.06	7.84	8.89	7.84	55.11	8.21	6.66	13.96	8.96	17.32	100.19	36.19	11.54	9.97	5.21	6.14	4.21	10.60	8.52	7.81
**Standard deviation**	6.02	1.90	2.16	2.50	2.65	2.42	7.63	3.47	1.76	2.66	2.28	2.73	14.26	5.86	2.63	3.10	1.65	1.70	1.12	2.88	2.56	2.41

MP-C: Models protection-composite; MP-H: Caregivers’ expectations for the adolescents’ academic achievement; MP-P: Peer disapproval of deviant behavior; MP-S: Classmates’ disapproval of use of drugs at school; MP-NO: Adults opinion of deviant behavior and use of drugs/alcohol; MP-NA: Adults in the neighborhood reactions to social deviance; CP-C: Control Protection-Component; CP-D: Adolescents’ judgment on the detrimental effects of drugs on health; CP-B: Adolescent’s beliefs about health behaviors; CP-H: Caregivers monitoring at the foster home center; CP-P: Peers’ sanctions for deviant behavior; CP-T: Teachers’ sanctions for deviant behavior;  SP-C: Support protection-Composite; SP-AD: Adolescents’ beliefs about antisocial behavior, smoking and use of illicit drugs; SP-AS: Adolescents’ positive attitude toward school; SP-HS: Caregivers support; SP-HM: Caregivers’ monitoring; SP-PS: Caregivers’ encouragement for prosocial behavior; SP-P: Peer support; SP-S: Caring and nurturing social climate at school; SP-I: Adolescents’ feeling of intimacy and connectedness with teachers/adults; SP-NE: Adolescents’ perceived neighborhood efficacy; R-1: Personal relationship with God; R-2: Factor 2 adolescent religiosity; R-3: Adolescents’ opinion about conventional religious motivated social practices; SDQ: Strengths and difficulties questionnaire; TD: Total difficulties; CP: Conduct problems; ADHD: Attention deficient and hyperactivity disorder; EP: Emotional problems; PP: Peer problems; PS: Prosocial behavior

* p < .05;

** p < .01.

**Table 2:** Hierarchical regressions of Strengths and Difficulties Questionnaire (SDQ) prosocial behavior on protective factors subscales (n = 140)

		**Total Sample (N = 140)**				**Females (n = 69)**				**Males (n = 71)**			
**Step**		**β** † **Step 2**	**B** ‡ ** Final** **Step**	**ΔR** ^2^	**R** ^2^	**β** † **Step 2**	**B** ‡ **Final Step**	**ΔR** ^2^	**R** ^2^	**β** † **Step 2**	**B** ‡ **Final Step**	**ΔR** ^2^	**R** ^2^
**1**	**Demographic Variables**			0.069***	0.069***			0.047	0.047			0.022	0.022
	**Gender**	-0.261***	-3.198‡‡										
	**Age**												
	**Home**												
**2**	**Protective Factors**			0.313***	0.382***			0.621***	0.668***			0.263	0.284
	**SP-AD**	0.256***	0.244‡			0.307*	0.192‡‡						
	**MP-H**					0.378**	1.09*						
	**MP-NO**					-0.364**	0.502‡‡						
**3**	**Protective Factors Interactions**			0.021	0.403			0.040	0.648			0.023	0.308
**4**	**Gender Interactions**			0.089	0.492								

SP-AD: Adolescents’ beliefs about antisocial behavior, smoking and use of illicit drugs; MP-H: Caregivers’ expectations for the adolescents’ academic achievement; MP-NO: Adults' opinion of deviant behavior and use of drugs/alcohol.

† Standardized regression weights at Step 3, before interaction terms are entered

‡ Unstandardized regression weights are displayed; standardized weights are deemed inappropriate with interaction terms (see Aiken &West, 1991, pp. 40–47).

§ Only significant interactions are included;

‡ Approaches significance;

‡‡ Non Significant

* p ≤ 0.05;

** p ≤ 0.001;

*** p ≤ 0.001

**Table 3:** Hierarchical regressions of Strengths and Difficulties Questionnaire (SDQ) difficulties scales on protective factors subscales (n = 140)

		**Conduct Problems**				**ADHD**				**Emotional Problems**						**Peer Problems**			
**Step**		**β** † **Step 2**	**B** †† **Final Step**	**ΔR** ^2^	**R** ^2^	**β** † **Step 2**	**B** †† **Final Step**	**ΔR** ^2^	**R** ^2^		**β** † **Step 2**	**B** †† **Final Step**	**ΔR** ^2^	**R** ^2^	**β** † **Step 2**		**B** †† **Final Step**	**ΔR** ^2^	**R** ^2^
**1**	**Demographic Variables**			0.014	0.014			0.054*	0.054*				0.013	0.013				0.010	0.010
	**Gender**					-0.178*	-3.98‡‡												
	**Age**																		
	**Home**																		
**2**	**Protective Factors**			0.256*	0.270*			0.139	0.193				0.233	0.246				0.172	0.182
	**R-3**	-0.218**	-0.134‡																
	**Protective Factors Interactions**			0.027	0.297			0.019	0.212				0.002	0.248				0.028	0.210
	**Gender Interactions**			0.175*	0.472 *			0.136	0.348				0.193*	0.441*				0.105	0.314
	**G x SP-AS**		-0.458*									-0.536**							
	**G x SP-I**		0.448*									0.463*							
	**G x MP-P**		-0.518*									0.434*							
	**G x CP-B**											-0.556*							

ADHD: Attention deficient and hyperactivity disorder; MR-H: Foster home models for risk behavior; R-3: Adolescents’ opinion about conventional religious motivated social practices; SP-AS: Adolescents’ positive attitude toward school; SP-I: Adolescents’ feeling of intimacy and connectedness with teachers/adults; MP-P: Peer disapproval of deviant behavior; CP-H: Caregivers monitoring at the foster home center; CP-B: Adolescent’s beliefs about health behaviors.

† Standardized regression weights at Step 3, before interaction terms are entered;

†† Unstandardized regression weights are displayed; standardized weights are deemed inappropriate with interaction terms (see Aiken &West, 1991, pp. 40–47).

††† Only significant interactions are included;

‡ Approaches significance (lower than p ≤ 0.10);

‡‡ Non Significant

* p ≤ 0.05;

** p ≤ 0.001;

*** p ≤ 0.001

**Table 4:** Hierarchical regressions of female Strengths and Difficulties Questionnaire (SDQ) difficulties scales on protective factors subscales (n = 69)

		**Conduct Problems**				**ADHD**				**Emotional Problems**				**Peer Problems**			
**Step**		**β** † **Step 2**	**B** †† **Final Step**	**ΔR** ^2^	**R** ^2^	**β** † **Step 2**	**B** †† **Final Step**	**ΔR2**	**R** ^2^	**β** † **Step 2**	**B** †† **Final Step**	**ΔR** ^2^	**R** ^2^	**β** † **Step 2**	**B** †† **Final Step**	**ΔR** ^2^	**R** ^2^
**1**	**Demographic Variables**			0.118***	0.118***			0.058‡	0.058‡			0.004	0.004			0.001	0.001
	**Age**	-0.316***	-0.386***														
	**Home**																
**2**	**Protective Factors**			0.556***	0.674***			0.358	0.415			0.647***	0.650***			0.464	0.465
	**R-1**	-0.366***	-0.243*							0.553***							
	**SP-AS**	0.539***	0.445‡							0.553***	0.649***						
	**SP-HM**	0.343***	0.412‡														
	**SP-PS**	-0.681***	-0.801***														
	**SP-AD** ^†††^									0.439***	0.326*						
	**CP-H**									-0.451***	-0.607***						
	**MP-P**									-0.499***	0.521‡‡						
	**Protective Factors Interactions**			0.010	0.683			0.121*	0.536*			0.102***	0.867***			0.009	0.474
	**MC x CC**						-0.002**										
	**MC x SC**										-.0009***						

ADHD: Attention deficient and hyperactivity disorder; R-1: Personal relationship with God; SP-AS: Adolescents’ positive attitude toward school; SP-HM: Caregivers’ monitoring; SP-PS: Caregivers’ encouragement for prosocial behavior; SP-AD: Adolescents’ beliefs about antisocial behavior, smoking and use of illicit drugs; CP-H: Caregivers monitoring at the foster home center; MP-P: Peer disapproval of deviant behavior; MC: Models protection composite index; CC: Control protection composite index; SC: Support protection composite index.

† Standardized regression weights at Step 3, before interaction terms are entered.

†† Unstandardized regression weights are displayed; standardized weights are deemed inappropriate with interaction terms (see Aiken &West, 1991, pp. 40–47).

††† Only significant interactions are included;

‡ Approaches significance (lower than p ≤ 0.10);

‡‡ Non Significant

* p ≤ 0.05;

** p ≤ 0.001;

*** p ≤ 0.001

Of interest, is the fact that in the case of females the protective factors predictive of conduct problems were different from those predicting emotional symptoms? Adolescents’ positive attitude toward school unexpectedly predicted both conduct problems and emotional symptoms. This finding can be explained within findings from previous studies regarding the fact that in case of maltreated and neglected youth, a high level of social support from peers and perceived social acceptance were positively correlated with engagement in various risk behaviors (21).

Possible explanations through which the relationship between some protective factors—like presence of caregivers encouragement for the participation of adolescents in profitable activities, such as playing different kinds of sports, and studying, as well as encouragement to eat healthy foods, behave correctly, and fasten seat belt when in an automobile—and prevention of conduct problems may be found in mechanisms associated with prosocial behavior, such as emotional regulation, social competence, and moral reasoning (22). With respect to the finding about positive prediction of conduct problems by caregivers’ monitoring, one possible interpretation could be that caregivers’ monitoring may be interpreted by the adolescent as a form of interference or intrusion and as such it represents a threat to his/her autonomy and integrity leading to oppositional and antisocial behaviors. A process similar to parent-child conflict as conceptualized in dynamic systems theory (23) to explain children’s antisocial development.

Among the predictors of emotional symptoms in females, besides adolescents’ positive attitude toward school which was addressed above, attitudinal intolerance of deviance was a positive predictor. As mentioned before, it has been found that one of the strongest predictors of risk behavior was this individual-level protection measure—attitudinal intolerance of deviance (15). The present findings indicate that as scores on this variable increase, emotional symptoms also increase. Future research is warranted to find out whether the present results are due to measurement problems or there is some empirical truth to it. However, the predictive association between this protective factor and prosocial behavior poses a challenge for future research.

Caregivers’ monitoring at the foster home and peer disapproval of deviant behavior were negative predictors of emotional symptoms (as in the case of females’ total difficulties), denoting a protective function preventing emotional symptoms. Of relevance to the interpretation of this finding is the fact that this same factor operated as a risk factor for females conduct problems. The differential association of caregivers’ monitoring has been recognized in the literature as an important protective factor (24) with externalizing and internalizing symptoms is of theoretical and practical significance. This supports the interactive and dynamic nature of the relationship of risk and protection with respect to adolescents’ problem behavior as postulated by different theories (15, 25). Considering the plasticity of individual-risks/protective factors relationships, and the possibility of different disorders co-existing in one individual, as co-morbidity is well known in the case of females (26), the identification of protective factors would profit from an integrative approach whereby not only risk and protective factors are simultaneously considered, but profiles of risk are constructed based on individual typologies (26).

To conclude, although the clinical research literature supports that many of the factors that place boys and girls involved in foster care, at risk for psychiatric problems are the same (27), the present findings suggests that each sex may respond differently to protective factors, as suggested by ecologically oriented research (8). In support of this assertion, a previous research (28) reported that grade point average (GPA) was the most salient protective factor against violence perpetration in both genders, but family connectedness, school connectedness, and religiosity also provided significant protection against violence perpetration for girls only, and this finding is in agreement with previous research findings.

In sum, overall, the present findings provide preliminary support for the applicability of the Problem Behavior Model in an Iranian sample of orphan adolescents, living in social and cultural contexts very different from those samples with which this theoretical model has been developed and tested in the past.

***Limitations and Recommendations***

The present study is not without shortcomings and limitations. One issue that remains to be further investigated is the role of attachment (29). Also, future research can expand on the assessment of religiosity and include religious coping.

***Implications for Practice***

The findings of the present research can be useful for curriculum design and planning for orphan children and adolescents that address the developmental needs of these vulnerable children considering models, controls and support protection parameters. Future research is warranted to apply a conceptual framework derived from an ecological and developmental psychopathological model (30), designed to identify protective factors, which may be associated with children’s long-term outcomes.

## Authors' contributions

MA conceived and designed the study, helped to draft the manuscript and revised the manuscript. MR prepared the research instruments, collected data, and was involved in interviews with caregivers, teachers and adolescent participants in the study. HRH participated in analyzing the data. All authors read and approved the final manuscript.

## Footenote

**Declaration of interest:** None.
